# The Effect of Adding Dexmedetomidine to Modified Del Nido Cardioplegia Solution on Myocardial Protection in Children Undergoing Congenital Heart Defect Surgery - A Prospective, Randomized, Triple-Blind, Controlled Trial

**DOI:** 10.21470/1678-9741-2025-0415

**Published:** 2026-09-08

**Authors:** Farhad Samadieh, Mahmood Hosseinzadeh Maleki, Masoud Razeghian, Tahereh Izanlou, Mohammadreza Naghibi Sistani, Benyamin Fazli, Mohsen Yaghubi

**Affiliations:** 1 Department of Extra-Corporeal Circulation (ECC), Imam Reza Hospital, Mashhad University of Medical Sciences, Mashhad, Iran; 2 Department of Cardiac Surgery, Imam Reza Hospital, Mashhad University of Medical Sciences, Mashhad, Iran; 3 Department of General Surgery, School of Medicine, Torbat Heydariyeh University of Medical Sciences, Torbat Heydariyeh, Iran; 4 Pediatric and Congenital Cardiology Division, Department of Pediatrics, Faculty of Medicine, Mashhad University of Medical Sciences, Mashhad, Iran; 5 Department of Anesthesiology, Faculty of Medicine, Mashhad University of Medical Sciences, Mashhad, Iran; 6 Department of Extra-Corporeal Circulation (ECC), Razavi Hospital, Imam Reza International University, Mashhad, Iran

**Keywords:** Dexmedetomidine, Del Nido Cardioplegia, Myocardial Protection, Congenital Heart Defects, Pediatric Cardiac Surgery.

## Abstract

**Introduction:**

The myocardial ischemia-reperfusion injury (IRI) is an indispensable factor in pediatric congenital heart defect (CHD) surgery. Although effective myocardial protection strategies are crucial, the issue remains a dilemma. This study aimed to evaluate the effect of adding dexmedetomidine to modified Del Nido cardioplegia on myocardial protection in children undergoing CHD surgery.

**Methods:**

This was a prospective, randomized, triple-blind, controlled clinical trial. A total of 60 pediatric patients were enrolled and randomized 1:1 into two groups - an intervention group that received dexmedetomidine-enriched modified Del Nido cardioplegia and a control group that received standard modified Del Nido cardioplegia with normal saline. Primary outcomes included serum troponin I and creatine kinase-MB levels, as well as coronary sinus lactate levels. Secondary outcomes were the vasoactive-inotropic score, weaning time from mechanical ventilation, and intensive care unit length of stay.

**Results:**

Adding dexmedetomidine to modified Del Nido cardioplegia significantly improved myocardial protection in children undergoing CHD surgery. The intervention group showed significantly lower troponin I levels at all postoperative time points compared to the control group (P < 0.001). Similarly, the dexmedetomidine group had significantly lower vasoactive-inotropic score at all time points (P < 0.001). Patients in the intervention group also experienced a significantly shorter mean ventilator weaning time (P < 0.001) and intensive care unit stay duration (P < 0.001). The overall distribution of postoperative morbidities did not differ significantly between the groups (P = 0.059).

**Conclusion:**

Adding dexmedetomidine to modified Del Nido cardioplegia significantly improved myocardial protection in children undergoing corrective CHD surgery.

## INTRODUCTION

**Table:** 

Abbreviations, Acronyms & Symbols				
AKI	= Acute kidney injury		IQR	= Interquartile range
ASD	= Atrial septal defect		IRI	= Ischemia-reperfusion injury
BMI	= Body mass index		Pulm.	= Pulmonary
CHD	= Congenital heart defect		RACHS	= Risk Adjustment for Congenital Heart Surgery
CK-MB	= Creatine kinase-MB		SD	= Standard deviation
CPB	= Cardiopulmonary bypass		VIS	= Vasoactive-inotropic score
HTN	= Hypertension		VSD	= Ventricular septal defect
ICU	= Intensive care unit			

Congenital heart defect (CHD) surgery in children often involves cardiopulmonary bypass (CPB), which can lead to myocardial ischemia-reperfusion injury (IRI)^[1-3]^. This injury significantly contributes to postoperative morbidity and mortality in pediatric patients^[1]^. Elevated serum troponin, a biomarker of myocardial damage, reflects direct trauma, inflammation, and ischemia, and has prognostic value^[1]^. Effective myocardial protection strategies are crucial to improve outcomes by addressing the multiple pathways of injury^[4,5]^.

Cardioplegia is essential for intraoperative cardioprotection, inducing electromechanical arrest to shield the myocardium from IRI^[1,6]^. It reduces metabolic demand and maintains cellular homeostasis^[5,7]^. Despite its importance, there is no universal consensus on the optimal cardioplegia solution^[1,5,6]^. Modified Del Nido cardioplegia, a four-part crystalloid-to-one-part whole-blood solution has been widely used in pediatric cardiac surgery for over 18 years^[7]^. Its single-dose administration, effective for up to two hours of aortic cross-clamping time, streamlines surgery and may reduce CPB duration^[7,8]^. Mechanistically, modified Del Nido induces a hyperpolarized arrest, preventing intracellular sodium and calcium accumulation, and includes trace amounts of calcium^[7,8]^. Clinical studies, particularly meta-analyses in adults, show modified Del Nido reduces myocardial injury markers and optimizes bypass times, confirming its efficacy^[8]^. Dexmedetomidine, an α2-adrenergic receptor agonist, is known for its sedative, analgesic, and anxiolytic properties^[9,10]^. Increasingly, it has been recognized for cardioprotective and anti-inflammatory effects against IRI^[10]^. Dexmedetomidine reduces myocardial inflammation by downregulating pro-inflammatory factors, such as tumor necrosis factor-α and interleukin-6^[10]^. Meta-analyses indicate dexmedetomidine is an effective cardioprotective agent in both adult and pediatric cardiac surgery, improving hemodynamic stability, reducing ventilation times, and lowering inotrope requirements^[9]^. In pediatric patients undergoing CPB, systematic reviews suggest dexmedetomidine can lower cardiac injury biomarkers (cardiac troponin I, creatine kinase-MB [CK-MB]) and improve clinical outcomes^[11]^. Combining modified Del Nido cardioplegia and dexmedetomidine has a strong scientific rationale, given their complementary mechanisms: modified Del Nido focuses on metabolic arrest and ionic homeostasis^[5,6]^, while dexmedetomidine targets inflammation and stress responses. This complementarity suggests potential synergistic benefits for comprehensive IRI mitigation. However, high-quality randomized controlled trials specifically evaluating this combined effect in children undergoing CHD surgery are scarce^[6]^. The existing pediatric cardioplegia literature often suffers from small sample sizes, heterogeneity, and a high risk of bias, which limits the ability to draw definitive conclusions^[3]^. This study aims to address this critical knowledge gap by providing robust evidence on this novel combined therapeutic strategy. Given the special vulnerability of the pediatric myocardium to IRI, this study aimed to evaluate the impact of adding dexmedetomidine to modified Del Nido cardioplegia on myocardial protection in children undergoing corrective surgery for CHDs.

## METHODS

### Study Design and Setting

This study was a prospective, randomized, triple-blind, controlled clinical trial conducted at three tertiary pediatric cardiac surgery centers in Iran: Imam Reza Cardiac Surgery Center (Mashhad), Razavi Cardiac Surgery Center (Mashhad), and Shahid Rajaei Cardiac Surgery Center (Tehran). The trial was designed to evaluate the effect of adding dexmedetomidine to modified Del Nido cardioplegia solution on myocardial protection and perioperative outcomes in children undergoing corrective surgery for CHDs. The sample size was calculated using the formula:

n = [(Z_1_-α/_2_ + Z_1_-β)^2^ × (σ_1_2 + σ_2_2)] / (µ1 - µ_2_)^2^

Where α = 0.05, β = 0.2 (power = 80%), and the expected means and standard deviations (SDs) for troponin-I were derived from a previous study^[11]^ (µ₁ = 1.03, µ₂ = 1.60, σ₁ = 0.55, σ₂ = 0.92). It resulted in a required sample size of 27.77 per group, which was rounded up to 30 per group to account for a 10% attrition rate.

### Ethics Approval and Consent to Participate

The Ethics Committee of Mashhad University of Medical Sciences, Mashhad, Iran (IR.MUMS.IRH.REC.1402.208) approved the study protocol. All procedures performed in this study were in accordance with the ethical standards of the institutional and/or national research committee, as well as the 1964 Declaration of Helsinki and its subsequent amendments or comparable ethical standards. Written informed consent was obtained from all subjects' guardians.

### Trial Registration

This study was retrospectively registered on the Iranian Registry of Clinical Trials (IRCT20240415061492N1).

### Participants

Eligible participants included pediatric patients older than one month scheduled for elective corrective surgery for CHDs with Risk Adjustment for Congenital Heart Surgery (RACHS) 1 or 2 complexity levels. To ensure comparability in baseline surgical risk between groups, enrollment was restricted to patients undergoing surgery classified within RACHS categories 1 or 2. This criterion was applied to mitigate confounding by significant differences in procedural complexity. Exclusion criteria were refusal of consent, significant liver dysfunction (defined as transaminase levels > 1.5× baseline), known congenital renal disease, coagulopathy, RACHS > 2, anticipated CPB or aortic cross-clamping times exceeding 120 minutes, complex surgical procedures, intraoperative death, confirmed postoperative sepsis (procalcitonin > 0.5 ng/ml with clinical signs), and refractory postoperative fever.

Patients were randomized 1:1 to intervention and control groups using computer-generated block randomization. Sealed opaque envelopes ensured allocation concealment. The study maintained triple blinding: patients' families, perfusionists, surgeons, anesthesiologists, intensive care unit (ICU) teams, and data analysts were all blinded to the group allocation.

### Intervention

All patients underwent standard midline sternotomy and CPB with systemic heparin anticoagulation (350 IU/kg, targeting an activated clotting time > 480 seconds), non-pulsatile roller-pump perfusion (2 - 2.8 L/min/m^2^), and mild hypothermia (32 - 34 °C). Arterial blood gases were managed using the alpha-stat strategy.

Antegrade cardioplegia was delivered after aortic cross-clamping via the coronary ostia. A modified Del Nido cardioplegia solution was prepared using isotonic saline 0.9% as the base, supplemented per liter with 16 ml of 20% mannitol, 4 ml of 50% magnesium sulfate, 13 ml of 7.5% sodium bicarbonate, 13 ml of 15% potassium chloride, and 6.5 ml of 2% lidocaine. This solution was mixed with autologous blood in a 4:1 ratio and administered at a dose of 20 ml/kg via the aortic root. Repeat half-volume doses were administered after 90 minutes if required.

For the intervention group, dexmedetomidine was added to the cardioplegia solution at a final concentration of 0.1 µg/mL (prepared by adding 2 mL of 50 µg/mL dexmedetomidine to 500 mL of cardioplegia solution). The control group received the same solution with an equivalent volume of normal saline. Cardioplegia administration continued until full electromechanical arrest was achieved.

Anesthesia was induced with fentanyl (10 - 15 µg/kg), midazolam (0.1 mg/kg), atracurium (0.5 mg/kg), and propofol (1.5 - 2 mg/kg). Maintenance included propofol infusion (50 - 100 µg/kg/min) and fentanyl (2 - 3 µg/kg/min). Intraoperative cerebral oximetry (near-infrared spectroscopy) monitoring was applied for all patients. Postoperatively, patients were transferred to the ICU, with extubation criteria including hemodynamic stability, normothermia, adequate respiratory effort, and acceptable blood gas values.

### Outcomes

The primary outcomes were serum troponin I levels, CK-MB, and coronary sinus lactate concentrations, measured at predefined time points. Troponin I and CK-MB were assessed at four time points: baseline (before surgery), upon ICU admission, and at 12 and 24 hours after ICU admission. The coronary sinus was cannulated with the blind technique. The correct positioning was confirmed by observing distension of the posterior interventricular vein, maintenance of coronary sinus pressure, and palpation of the coronary sinus cannula. Coronary sinus lactate was measured intraoperatively at three points: immediately after opening the right atrium, just before aortic cross-clamp removal, and ten minutes after declamping. Secondary outcomes included the vasoactive-inotropic score (VIS), calculated as dopamine + dobutamine (µg/kg/min) × 1 + milrinone × 15 + (epinephrine + norepinephrine + isoproterenol) × 100 dopamine + dobutamine (µg/kg/min) × 1 + milrinone × 15 + (epinephrine + norepinephrine + isoproterenol) × 100 and assessed at ICU admission and at six, 24, and 48 hours postoperatively; the incidence of acute kidney injury (AKI), defined as an increase in serum creatinine of ≥ 0.3 mg/dL or > 50% from baseline; the duration of mechanical ventilation (time to extubation); and length of stay in the ICU.

Additionally, the following clinical and demographic data were collected: age, sex, weight, height, cardiac diagnosis, total cardioplegia volume administered, aortic cross-clamping time, and total CPB time. Postoperative morbidities, including neurological complications (seizure, coma, cerebral edema, or documented brain injury based on imaging), arrhythmias, pulmonary congestion, pneumonia, and jaundice, were also recorded. Postoperative arrhythmias were recorded based on clinical documentation and electrocardiogram monitoring. Specific arrhythmia types were not systematically classified in this study.

### Statistical Analysis

Statistical analyses were performed using IBM SPSS Statistics for Windows, version 27.0 (IBM Corp., Armonk, N.Y., USA). Continuous variables were expressed as mean ± SD for normally distributed data or as median with interquartile range for non-normally distributed data. Categorical variables were presented as frequencies and percentages.

Normality of continuous data was assessed using the Kolmogorov-Smirnov test. Between-group comparisons of continuous variables were conducted using the independent *t*-test or the Mann-Whitney U test, as appropriate. Categorical variables were compared using the chi-square test or Fisher's exact test.

Repeated measures analysis of variance or the Friedman test was applied for intragroup comparisons of continuous variables measured at multiple time points. A Bonferroni correction was used for post hoc multiple comparisons. A two-sided *P*-value of < 0.05 was considered statistically significant. All data were coded and analyzed by a biostatistician who was blinded to group assignments.

## RESULTS

A total of 60 pediatric patients undergoing corrective surgery for CHDs were enrolled in our study. Patients were randomly assigned to two groups: the intervention group, which received dexmedetomidine-enriched modified Del Nido cardioplegia (n = 30), and the control group, which received standard modified Del Nido cardioplegia with normal saline (n = 30). All analyses were conducted on an intention-to-treat basis.

### Baseline and Intraoperative Characteristics

Baseline demographics and intraoperative variables were comparable between groups, with no statistically significant differences in age, sex distribution, body mass index, aortic cross-clamping time, or CBP duration (Table 1). This balance ensured that any observed postoperative differences could be attributed to the intervention rather than underlying differences in patient characteristics. The distribution of CHDs was also similar across groups (*P* = 0.384), supporting clinical comparability (Figure 1).

**Table 1:** Baseline demographics and intraoperative characteristics of study groups.

Characteristic	Dexmedetomidine (n = 30)	Control (n = 30)	*P*-value
Sex			
Male, n (%)	14 (46.7%)	15 (50.0%)	0.796
Female, n (%)	16 (53.3%)	15 (50.0%)	
Age (months), mean ± SD	33.9 ± 24.58	38.10 ± 41.44	0.300
BMI (kg/m^2^), mean ± SD	10.59 ± 3.08	10.49 ± 2.94	0.902
Aortic cross-clamping time (min), mean ± SD	76.00 ± 22.15	73.17 ± 7.09	0.509
CPB time (min), mean ± SD	92.50 ± 24.56	85.73 ± 7.67	0.159

BMI=body mass index; CPB=cardiopulmonary bypass; SD=standard deviation

**Fig. 1:**
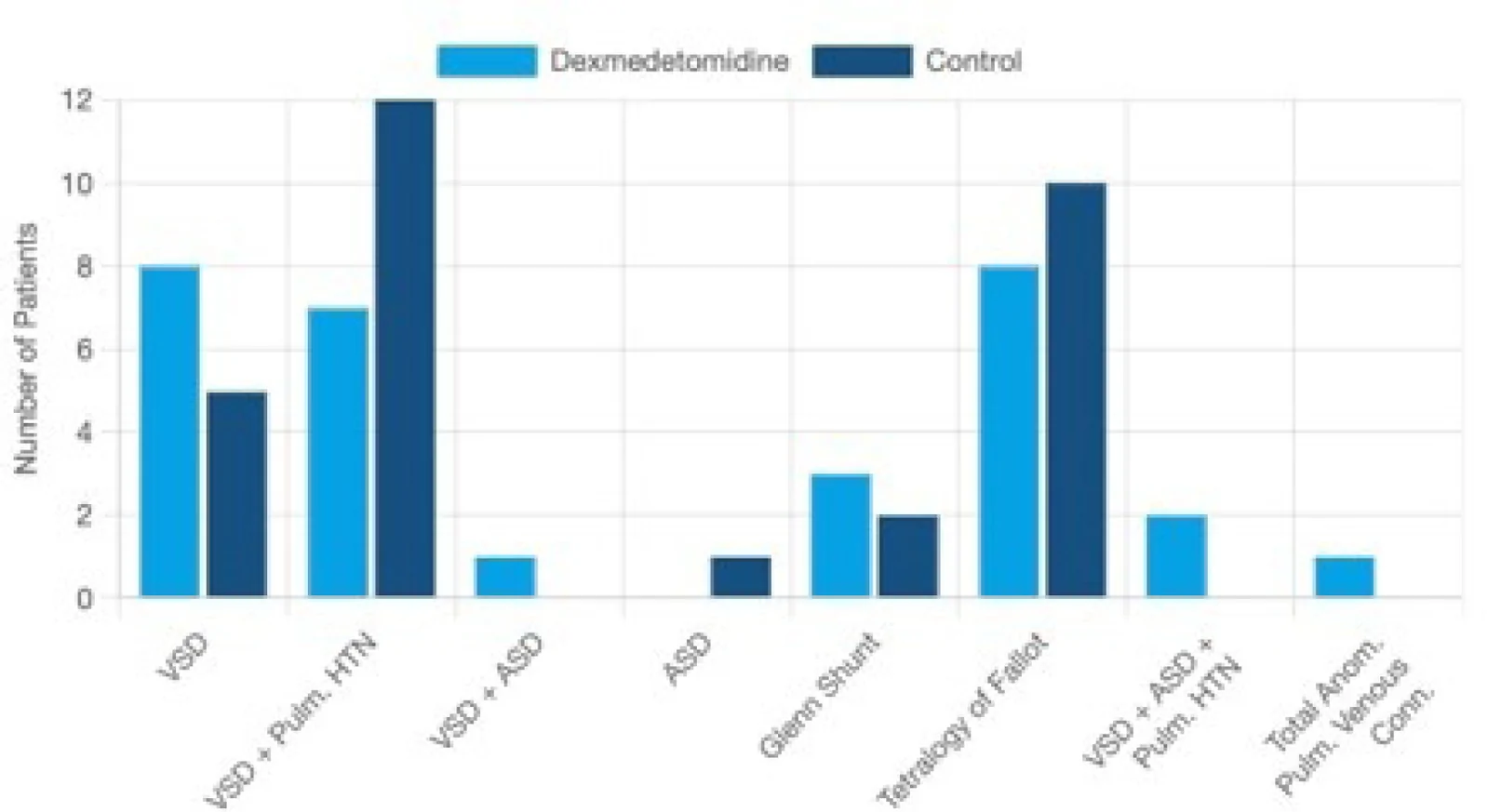
Distribution of congenital heart defects. ASD=atrial septal defect; HTN=hypertension; Pulm=pulmonary; Total Anom. Pulm. Venous Conn=total anomalous pulmonary venous connection; VSD=ventricular septal defect.

### Coronary Sinus Lactate

As presented in Table 2, at baseline (T0), mean coronary sinus lactate levels were significantly higher in the dexmedetomidine group compared with the control group (*P* < 0.0001). This difference remained statistically significant immediately before removal of the aortic cross-clamp (T1) (*P* < 0.001). However, ten minutes after cross-clamp removal (T2), lactate levels were comparable between groups (*P* = 0.638), indicating no statistically significant difference at this time point.

**Table 2:** Comparison of coronary sinus lactate levels (mmol/L) between the dexmedetomidine and control groups, at different perioperative time points.

Time point	Dexmedetomidine (mean ± SD)	No dexmedetomidine (mean ± SD)	*t*-statistic	*P*-value
T0	5.66 ± 1.43	3.26 ± 0.69	8.28	< 0.0001
T1	6.75 ± 1.32	5.52 ± 1.25	3.71	< 0.001
T2	6.61 ± 1.41	6.77 ± 1.20	-0.47	0.638

SD=standard deviation

Note: *P*-values were calculated using independent samples *t*-tests. T0: immediately after opening the right atrium; T1: immediately before removal of the aortic cross-clamp; T2: ten minutes after removal of the aortic cross-clamp

### Myocardial Injury Markers and Clinical Outcomes

Analysis of myocardial injury biomarkers revealed significantly reduced postoperative elevations in the dexmedetomidine group (Table 3). For troponin I, median values increased postoperatively in both groups, with the highest levels observed at ICU admission and early postoperative hours. However, across all postoperative time points (ICU admission, 12 hours, and 24 hours), the dexmedetomidine group consistently showed lower median troponin I concentrations compared to the control group. CK-MB values followed a similar pattern. Both groups exhibited an initial rise after surgery, with measurements peaking at ICU admission and then declining thereafter. The dexmedetomidine group demonstrated lower median CK-MB levels at 12 hours and 24 hours postoperatively compared to the control group, while baseline and ICU admission values were comparable between groups. VIS values were recorded at ICU arrival and at six, 24, and 48 hours postoperatively. Both groups showed a decrease in VIS scores over time. However, the dexmedetomidine group consistently had lower median VIS scores at each measurement point compared to the controls.

**Table 3:** Myocardial injury markers.

Characteristic	Time point	Dexmedetomidine (median / IQR)	Control (median / IQR)	Inter-group *P*-value (Mann-Whitney U)	Intra-group *P*-value (Friedman test)
Troponin	T0	0.15 (0.070 - 0.19)	0.19 (0.14 - 0.23)	< 0.001	< 0.001 (dexmedetomidine) / < 0.001 (control)
	T1	0.19 (0.11 - 0.24)	0.29 (0.23 - 0.34)	< 0.001	
	T2	0.11 (0.08 - 0.17)	0.37 (0.31 - 0.40)	< 0.001	
	T3	0.08 (0.05 - 0.09)	0.47 (0.41 - 0.51)	< 0.001	
CK-MB	T0	55.0 (48 - 59)	45.5 (42 - 51)	0.055	< 0.001 (dexmedetomidine) / < 0.001 (control)
	T1	64.0 (53 - 73)	57.5 (52 - 62)	0.473	
	T2	51.25 (47 - 56)	65.5 (61 - 74)	< 0.001	
	T3	48.50 (45 - 55)	78.5 (74 - 85)	< 0.001	
VIS	T0	27.31 (24.25 - 30.12)	33.95 (30.5 - 35.5)	< 0.001	< 0.001 (dexmedetomidine) / < 0.001 (control)
	T1	23.68 (20.0 - 25.5)	29.21 (25.0 - 33.0)	< 0.001	
	T2	19.50 (15.37 - 20.50)	24.86 (20.50 - 28.87)	< 0.001	
	T3	8.57 (5.00 - 11.75)	18.83 (15.00 - 25.50)	< 0.001	

CK-MB=creatine kinase-MB; ICU=intensive care unit; IQR=interquartile range; VIS=vasoactive-inotropic score

Note: Values are median / IQR unless otherwise specified. T0: before the surgery; T1: upon ICU admission; T2: 12 hours post-ICU admission; T3: 24 hours post-ICU admission

These time-based trends and intergroup differences in troponin I, CK-MB, and VIS are presented in Table 2 and illustrated in Figure 2.

**Fig. 2:**
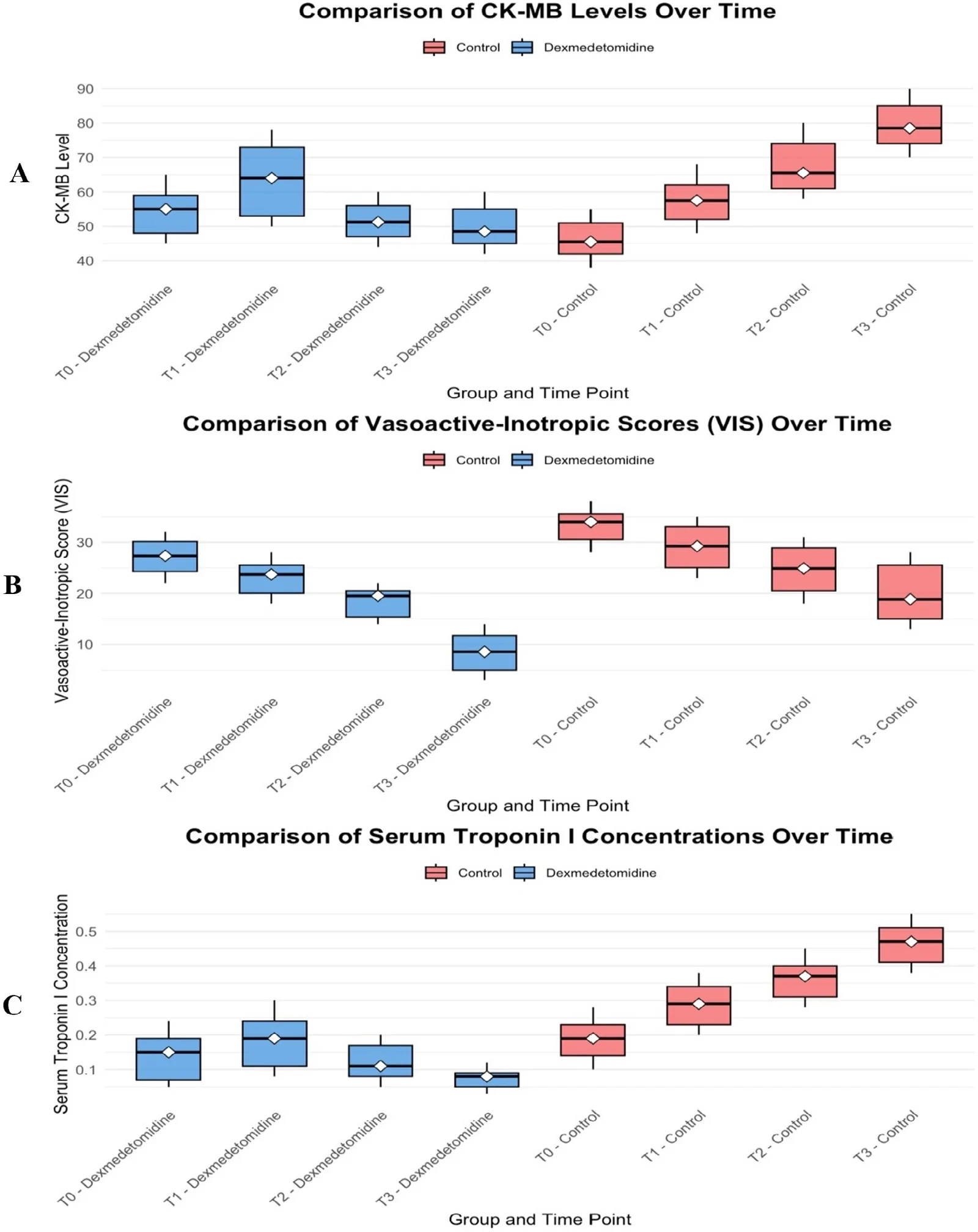
Perioperative measurements of myocardial injury markers and hemodynamic support requirements. Creatine kinase-MB (CK-MB) levels (A), vasoactive-inotropic scores (B), and serum troponin I concentrations (C) are plotted over time for both groups. Data are presented as median with interquartile ranges. T0: Preoperation; T1: post intensive care unit (ICU) admission; T2: 12 hours post ICU admission; T3: 24 hours post ICU admission.

Analysis of myocardial injury biomarkers revealed attenuated postoperative elevations in the dexmedetomidine group compared to the control group. Figure 2 summarizes these trends, showing that serum troponin I and CK-MB levels remained consistently lower at all postoperative time points in the intervention group, suggesting reduced myocardial cellular injury and superior cardioprotection.

Additionally, the dexmedetomidine group exhibited consistently lower VIS throughout the perioperative period. This reduction in pharmacologic support requirements indicates improved hemodynamic stability and reduced myocardial stress during recovery.

### Postoperative Parameters

Table 4 presents postoperative clinical outcomes for both groups. The mean weaning time from mechanical ventilation was shorter in the dexmedetomidine group compared to the control group. Similarly, the duration of ICU stay was lower in the dexmedetomidine group.

**Table 4:** Postoperative clinical outcomes in dexmedetomidine and control groups.

Outcome	Dexmedetomidine group	Control group	*P*-value
Weaning time (min)	455.0 ± 64.9	624.5 ± 113.8	< 0.001
ICU stay duration (hours)	79.25 ± 24.67	120.96 ± 96.78	< 0.001

ICU=intensive care unit

Postoperative morbidities were also evaluated in all patients. As shown in Table 5, arrhythmia (junctional ectopic tachycardia in three cases and atrial tachycardia in one case) was the most frequent morbidity in the dexmedetomidine group (13.4%), while AKI was most common in the control group (23.3%). Other complications, such as pulmonary congestion, occurred at comparable rates in both groups. Overall, 80.0% of patients in the dexmedetomidine group and 66.7% in the control group experienced no postoperative morbidities. However, the distribution of postoperative morbidities between the two groups was not statistically significant (χ^2^ = 6.997, *P* = 0.059).

**Table 5:** Comparison of postoperative morbidities between the dexmedetomidine and control groups.

Postoperative morbidity	Dexmedetomidine group	Control group	Chi-square test
	N (%)	N (%)	
Arrhythmia	4 (13.4%)	1 (3.3%)
Pulmonary congestion	1 (3.3%)	2 (6.7%)
AKI	1 (3.3%)	7 (23.3%)
No morbidity	24 (80.0%)	20 (66.7%)
Total	30 (100%)	30 (100%)	χ^2^ = 6.997, *P* = 0.059

AKI=acute kidney injury

## DISCUSSION

Myocardial protection during cardiac surgery remains a cornerstone of optimizing surgical outcomes, especially in pediatric patients. Various strategies have been employed to improve myocardial preservation, including different cardioplegia solutions, perioperative pharmacologic agents, and innovative techniques such as terminal hot-shot infusions or the use of antioxidant opioids in the aortic root^[12]^. However, no universally accepted method has been established as both safe and highly effective in pediatric cardiac surgery.

The present study demonstrated that adding dexmedetomidine to modified Del Nido cardioplegia significantly reduced myocardial injury markers, such as troponin and CK-MB, at multiple postoperative time points compared to isotonic saline. These findings align with previous studies reporting decreased myocardial enzyme release after perioperative dexmedetomidine administration in both adult and pediatric cardiac surgeries^[13,14]^. Notably, Chi et al.^[13]^ conducted a retrospective cohort study of 1,134 adult cardiac surgery patients and reported that perioperative dexmedetomidine infusion was associated with substantial reductions in in-hospital mortality, 30-day mortality, and one-year mortality after risk adjustment.

An important strength of our study lies in the novel approach of administering dexmedetomidine directly in combination with cardioplegia. This method ensures localized myocardial exposure to a cardioprotective agent with anti-inflammatory properties while minimizing systemic side effects. Furthermore, dosing was calculated based on estimated coronary perfusion, which may explain the absence of significant bradycardia or persistent hypotension observed in other trials using higher systemic doses^[15]^.

Coronary sinus lactate levels showed an initial difference at T0 and T1, with the dexmedetomidine group having higher absolute values than the controls, likely reflecting baseline metabolic status or differences in myocardial substrate utilization prior to reperfusion. Importantly, by T2 (10 minutes post-declamping), lactate levels had equalized between groups, suggesting similar recovery of myocardial oxygen utilization after reperfusion. Therefore, while dexmedetomidine did not reduce absolute lactate levels during the ischemic phase, its role in biochemical recovery post-reperfusion remains plausible. This finding is consistent with evidence that dexmedetomidine can attenuate IRI, partly through anti-inflammatory and microcirculatory benefits^[16-18]^.

Another notable finding was a significant reduction in the VIS within the first 24 hours postoperatively in the dexmedetomidine group. A reduced need for vasoactive support may indicate better preserved myocardial contractility and hemodynamic stability^[19]^. While some studies have reported increased bradycardia and hypotension with dexmedetomidine^[20]^, our method of delivery via cardioplegia likely limited systemic exposure and avoided such adverse effects.

Interestingly, although the overall incidence of arrhythmias was higher in the dexmedetomidine group, these events occurred in patients with complex cardiac anomalies and severe pulmonary hypertension, which may confound interpretation. Other literature suggests that dexmedetomidine can reduce the risk of arrhythmias in pediatric cardiac populations^[21]^.

Ventilation time and ICU/hospital length of stay were significantly reduced in our intervention group, consistent with earlier randomized controlled trials and meta-analyses^[22]^. Shorter ventilation times are clinically relevant as they may lower the risk of ventilator-associated complications and reduce resource utilization. Furthermore, previous studies have shown that perioperative dexmedetomidine may decrease inflammatory lung injury during thoracic surgery^[23]^, supporting its role in enhancing recovery profiles.

### Limitations

Overall, our findings add to the growing body of evidence that dexmedetomidine, when integrated into cardioplegia, may provide synergistic myocardial protection, improve perioperative hemodynamics, and enhance postoperative recovery in pediatric cardiac surgery. However, certain limitations should be acknowledged. The sample size, although powered to detect differences in troponin I levels, limits the detection of rarer adverse events or nuanced subgroup effects. The study was conducted in specialized centers, which may limit generalizability to all surgical settings. Moreover, while short-term biochemical and clinical outcomes were assessed, longer-term follow-up was not included to evaluate sustained myocardial or renal function or late morbidity. Finally, although the study demonstrates biochemical and clinical benefits, the precise mechanistic pathways by which dexmedetomidine confers myocardial protection when added to cardioplegia warrant further investigation. Future multicenter randomized trials with larger sample sizes are warranted to confirm these benefits, evaluate long-term outcomes, and optimize dosing strategies for this novel administration route.

## CONCLUSION

The present study demonstrates that the addition of dexmedetomidine to modified Del Nido cardioplegia is a successful strategy for myocardial protection in children undergoing corrective surgery for CHDs. This method led to significantly lower postoperative elevations in myocardial injury biomarkers, such as troponin I and CK-MB, compared with the control group. The intervention also resulted in consistently lower VIS, suggesting better preserved myocardial contractility and improved hemodynamic stability. Furthermore, patients receiving dexmedetomidine-enriched cardioplegia experienced significantly shorter ventilator weaning times and a shorter ICU length of stay, indicating enhanced postoperative recovery and a potential reduction in resource utilization. While a higher incidence of arrhythmias was noted in the dexmedetomidine group, this difference was not statistically significant and may have been confounded by patient complexity.

## Data Availability

The authors declare that the data will be available upon reasonable request to the authors.

## Artificial Intelligence Usage

The authors confirms that no artificial intelligence tools or Large Language Models (LLMs) were used in the writing, analysis, or any other stage of this paper.
